# Incidental Myocardial Bridging Coinciding With Myocardial Ischemia and Takotsubo Cardiomyopathy in an Asymptomatic Patient

**DOI:** 10.7759/cureus.103663

**Published:** 2026-02-15

**Authors:** Muhammad Z Khan, Aadhar Patil, Mohamed Suleiman, Jade Karr

**Affiliations:** 1 Internal Medicine, St. Mary Medical Center, Langhorne, USA; 2 Cardiology, Jefferson Torresdale Hospital, Philadelphia, USA; 3 Internal Medicine, Jefferson Torresdale Hospital, Philadelphia, USA; 4 Internal Medicine, Thomas Jefferson University Hospital, Philadelphia, USA

**Keywords:** cardiomyopathy, myocardial bridging (mb), myocardial ischemia and infarction, st-segment elevation mi, takotsubo cardiomyopathy

## Abstract

Although most cases of myocardial bridging (MB) are clinically benign, it can sometimes be a risk factor for myocardial infarction (MI) and life-threatening arrhythmia. In this study, we present a case of MB-induced ST-segment elevation MI in a patient with stress cardiomyopathy. A 71-year-old man presented to the emergency department for evaluation of hepatic encephalopathy. The initial ECG was consistent with generalized ST elevation between leads 1, 2, 3, and V1-V6. Coronary angiography showed a segment of severe myocardial bridging in the mid-distal left anterior descending (LAD). Bridging caused almost complete beat-to-beat closure of the LAD, which was unchanged during coronary nicardipine administration. Myocardial bridging was treated medically with beta-blockers and calcium channel blockers. Echocardiography showed mild left ventricular systolic function deterioration, an ejection fraction of 35-40%, and apical ballooning consistent with stress cardiomyopathy. A repeat ECG several days later showed normal sinus rhythm and nonspecific T-wave abnormality. As our case report demonstrates, myocardial bridging and stress-induced cardiomyopathy can have significant clinical consequences and even manifest as acute coronary syndrome in certain clinical situations, which raises a compelling issue.

## Introduction

Myocardial bridging (MB) is a congenital variant of the coronary artery in which the vessel passes through the myocardium instead of the typical epicardial pathway [1]. Möhlenkam et al. mentioned that some anatomic features of MB, including length (≥2.5 cm), depth (≥2.0 mm), and severe systolic squeezing (≥70%), may be linked to the development of coronary artery disease (CAD) and atherosclerotic lesions, as well as subsequent major adverse cardiac events (MACE) [2]. Myocardial infarction (MI), arrhythmia, syncope, sudden cardiac arrest, stress-induced or hypertrophic cardiomyopathy, myocardium shocking, vasospasm angina, and decreased left ventricular functions in patients with MB have all been documented occurrences, with MI being the most commonly reported [1,3]. These complications occur when the tunneled artery is compressed during systole and the overlying muscle fibers and increased flow velocity cause stress and vascular damage [3]. In particular, coronary vasospasm (CVS) can act as a trigger in such situations in patients with MB [3]. There is limited data that shows that stress cardiomyopathy can cause MB. Lemaitre et al. suggested that myocardial bridging was possibly enhanced by catecholamines during stress in stress cardiomyopathy [4]. We present a case of ST-segment elevation myocardial infarction (STEMI) triggered by stress cardiomyopathy.

## Case presentation

**Figure 1:**
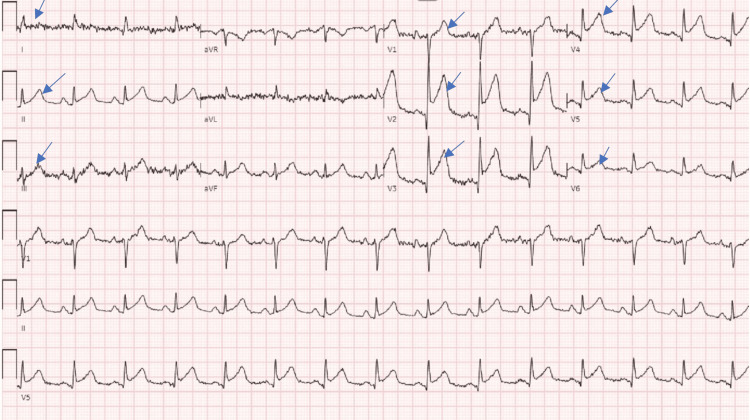
Arrows show widespread ST elevations across leads I, II, III, V1-V6.

A 71-year-old man was brought by ambulance from the nursing facility to the emergency department (ED) for an encephalopathy assessment. He had a significant medical history, including paroxysmal atrial fibrillation on apixaban, a history of seizures without medication, esophageal rupture repair, hepatitis C virus, cirrhosis due to hepatitis C virus, long-standing hypertension, and substance and alcohol use disorder. His home medications included apixaban 5 mg twice a day, amlodipine 5 mg daily, and omeprazole 40 mg daily. The patient had jaundice, bilateral nystagmus, confusion, disorientation, and a crescendo-decrescendo systolic murmur radiating into carotid arteries that were audible. During the initial arrival at the ED, his vitals were stable; the patient denied chest pain and shortness of breath. The initial electrocardiogram (EKG) was consistent with widespread ST elevations across leads I, II, III, and V1-V6, as illustrated in (Figure 1).

Remarkable laboratory findings are mentioned in Table 1. Ammonia level was 121 mcg/dL and bilirubin was 1.7 mg/dL.

**Table 1:** Initial laboratory evaluation obtained upon arrival.

Laboratory test	Value	Reference range
Creatinine	1.32 mg/dl	0.5-0.9 mg/dl
Glomerular filtration rate (GFR)	58 mL/min	> 60 mL/min
Alkaline phosphatase	181 IU/L	35-105 IU/L
Aspartate aminotransferase (AST)	60 IU/L	0-32 IU/L
Alanine aminotransferase (ALT)	37 IU/L	0-33 IU/L
Hs-troponin	157 ng/L	<5ng/L
Platelet count	420,000 µL	140,000-400,000 µL

He was given aspirin 325 mg. Despite denying chest pain, nausea, shortness of breath, or similar symptoms, his confusion raised questions about self-assessment reliability. Initial hs-troponin levels were at 157 ng/L (normal troponin < 5ng/L), which rose sharply to 1192 ng/L; in response, he was administered a heparin infusion, and the patient remained hemodynamically stable. An emergent cardiac catheterization was performed based on initial EKG findings and the significant increase in repeated troponin levels. Coronary angiography showed a segment of severe myocardial bridging causing near-complete occlusion of the mid to distal left anterior descending (LAD) during the full systolic cycle, which did not change with intracoronary nicardipine administration (Video 1). This was identified as the cause of his clinical presentation. Otherwise, there is only mild atherosclerotic coronary artery disease (CAD).

**Video 1:** Myocardial bridging. Coronary angiography showed a segment of severe myocardial bridging causing near complete occlusion of the mid to distal left anterior descending artery.

Aspirin and statin were continued. The myocardial bridging segment was treated medically with beta-blockers, metoprolol tartrate 25 mg twice daily, and calcium channel blockers, such as amlodipine 5 mg. Transthoracic echocardiography showed mildly decreased left ventricular systolic function, an ejection fraction of 35-40%, and segmental wall motion abnormalities. There is apical akinesis and ballooning of the left ventricular apex consistent with apical-type Takotsubo (stress-induced) cardiomyopathy (Video 2).

**Video 2:** Segmental wall motional abnormalities in myocardial bridging. Transthoracic echocardiography showed decreased left ventricular systolic function, apical akinesis, and ballooning of the left ventricular apex.

There was no spectral Doppler evaluation of the left ventricular outflow tract (LVOT), but there appears to be turbulence in the LVOT by color flow, suggesting dynamic obstructions. Hypotension was seen from LVOT obstruction but was improved with fluid resuscitation.

**Figure 2:**
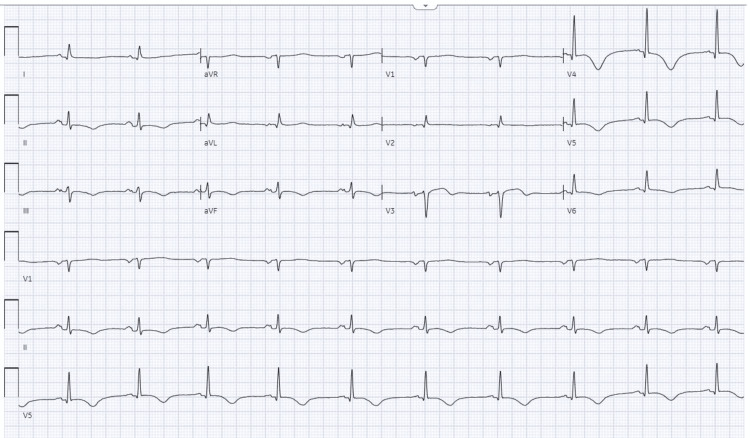
Show normal sinus rhythm, left atrial enlargement, and nonspecific T-wave abnormality.

His hospital course was complicated with atrial fibrillation with rapid ventricular response (RVR), which was treated with amiodarone (initially loaded with 400 mg three times a day and then switched to 200 mg daily), and the metoprolol dosage was increased from 25 mg to 50 mg twice daily (bid). The course was further complicated by sinus bradycardia (49 bpm) on amiodarone and metoprolol, reducing the metoprolol dose to 25 mg two times daily. The patient was a poor candidate for atrial fibrillation ablation mainly because of esophageal pathology and the high risk for cardio-esophageal fistula complications as well as co-morbidities. Repeated EKG after several days showed normal sinus rhythm, possible left atrial enlargement, nonspecific T wave abnormality, and prolonged QTc interval 504 milliseconds (ms) from a baseline of 450 ms in Figure 2. Amiodarone was stopped because of prolonged QTc.

The patient was discharged from the hospital. Echo after four months showed a recovered ejection fraction (EF) of 55%.

## Discussion

As our case report illustrates, the intertwining of incidental MB-inducing myocardial ischemia and Takotsubo cardiomyopathy in the absence of classic symptoms of these conditions underscores a compelling clinical conundrum. One hypothesis that could account for the acute presentation would be that stress cardiomyopathy resulted in hypercontractility of the base of the left ventricle. It was the LAD that coursed through the base of the anterior wall that was found to exhibit severe bridging.

Myocardial bridges are anatomical anomalies of a coronary artery running intramurally through the myocardium. If blood flow is sufficiently restricted, this may cause the "bridge" to compress during systole, potentially leading to myocardial ischemia. MB, usually considered benign, can lead to symptomatic manifestations including, but not limited to, chest pain, arrhythmias, and, in rare cases, sudden cardiac death [5].

In a retrospective study of 132 cases of sudden cardiac death, it was found that 21.2% had MB, primarily in the left anterior descending artery [6]. The prevalence of MB in the general population is variable, with autopsy studies reporting rates between 5-86%, while angiographic detection is much lower due to its transient nature, only observable during systole [7]. The variation in the prevalence may be due to regional variation, sampling method, or interobserver variation. Bridges with only fibrous roofs may also be easily damaged during dissection [7].

Based on recent studies, two mechanisms are responsible for MB: a phasic systolic vessel compression with persistent mid-to-late diastolic diameter reduction and increased intracoronary Doppler flow velocities with abnormal qualitative flow profiles [8]. Symptoms typically arise due to the compression of the tunneled arterial segment during systole, leading to temporary myocardial ischemia.

In this case report, the concomitant emergence of myocardial ischemia due to myocardial bridging resulted from hypercontractility of the base of the left ventricle from stress cardiomyopathy, which was in our patient, and is an unusual clinical finding, particularly with subclinical patient presentation and lack of conventional symptoms like chest pain, shortness of breath on exertion, or gastrointestinal discomfort, which were not noted during the initial presentation, besides confirmation with EKG and cardiac troponin, which all point to myocardial ischemia. The prompt performance of an emergency cardiac catheterization confirmed myocardial bridging, obviating the need to consider other possible diagnoses usually considered for such cases. The emergency cardiac catheterization validated the existence of myocardial bridging, thus excluding the necessity for differential diagnoses that typically arise in such asymptomatic patients.

The confluence of myocardial bridging-induced ischemia with stress-induced cardiomyopathy is noteworthy. Both conditions share myocardial stunning as a common pathophysiological mechanism, yet their clinical interplay in minimal CAD is scarcely documented [9]. Takotsubo cardiomyopathy, while transient, can manifest due to sudden hemodynamic shifts caused by unanticipated myocardial bridging-related arterial compression during systole [2,10].

Currently, clinicians need more agreement regarding the definitive treatment strategy for ischemia-inducing MB or including other complications induced by MB. Standard care ischemia-inducing MB involves medical therapy, including beta-blockers and calcium channel blockers, to more invasive approaches such as surgical myotomy or coronary artery bypass grafting (CABG) in refractory [11]. Our case report underscores the imperative of an integrated approach to successfully diagnose immediately with cardiac catheterization, succeeded by standard medical therapy, with beta-blockers and calcium blockers in specific patient categories. Invasive management was not done because our thought process was that stress cardiomyopathy resulted in hypercontractility of the base of the left ventricle, which exaggerated the MB. Patient EF recovered in four months.

## Conclusions

Myocardial bridging patients may experience syncope, myocardial ischemia, and sudden death. Our case report demonstrates that stress cardiomyopathy can manifest as an acute coronary syndrome. Stress cardiomyopathy results in hypercontractility of the base of the left ventricle, which can exaggerate the MB. These patients can be medically managed instead of having interventions. Clinicians should be vigilant for cardiac anomalies even in people without symptoms, acknowledging that coronary anomalies and related complications can merge in surprising manners.
